# Supplementary material for: Multi‐dataset identification of innovative feature genes and molecular mechanisms in keratoconus
Source: J Cell Mol Med. 2024 Sep 19;28(18):e70079. doi: 10.1111/jcmm.70079 (PMC11412914; doi:10.1111/jcmm.70079)
Supplement: Supplementary file 2 — Material S2. [file JCMM-28-e70079-s002.zip › 7.GSEA analysis/2.ATOH7/KEGG_MAPK_SIGNALING_PATHWAY.html]

Details for gene set KEGG\_MAPK\_SIGNALING\_PATHWAY[GSEA]

|  || Dataset | ATOH7.group.cls#h\_versus\_l |
| Phenotype | group.cls#h\_versus\_l |
| Upregulated in class | h |
| GeneSet | KEGG\_MAPK\_SIGNALING\_PATHWAY |
| Enrichment Score (ES) | 0.33700085 |
| Normalized Enrichment Score (NES) | 1.37157 |
| Nominal p-value | 0.05586592 |
| FDR q-value | 0.32523176 |
| FWER p-Value | 0.926 |
Table: GSEA Results Summary

  

Fig 1: Enrichment plot: KEGG\_MAPK\_SIGNALING\_PATHWAY      
 Profile of the Running ES Score & Positions of GeneSet Members on the Rank Ordered List

  

| SYMBOL | TITLE | RANK IN GENE LIST | RANK METRIC SCORE | RUNNING ES | CORE ENRICHMENT || 1 | AKT3 | na | 9 | 1.043 | 0.0285 | Yes |
| 2 | CACNA2D1 | na | 19 | 0.977 | 0.0553 | Yes |
| 3 | MRAS | na | 141 | 0.733 | 0.0720 | Yes |
| 4 | IL1R2 | na | 423 | 0.570 | 0.0796 | Yes |
| 5 | DUSP10 | na | 515 | 0.544 | 0.0919 | Yes |
| 6 | FGFR1 | na | 573 | 0.527 | 0.1048 | Yes |
| 7 | CACNA1G | na | 646 | 0.512 | 0.1169 | Yes |
| 8 | CACNA1B | na | 672 | 0.503 | 0.1300 | Yes |
| 9 | CACNA2D2 | na | 743 | 0.489 | 0.1415 | Yes |
| 10 | PRKACB | na | 829 | 0.474 | 0.1521 | Yes |
| 11 | RASGRP1 | na | 977 | 0.451 | 0.1603 | Yes |
| 12 | DDIT3 | na | 1043 | 0.441 | 0.1706 | Yes |
| 13 | RASGRP3 | na | 1283 | 0.410 | 0.1750 | Yes |
| 14 | NTRK2 | na | 1392 | 0.398 | 0.1828 | Yes |
| 15 | IL1R1 | na | 1408 | 0.396 | 0.1933 | Yes |
| 16 | CACNA2D4 | na | 1506 | 0.385 | 0.2011 | Yes |
| 17 | MEF2C | na | 1616 | 0.375 | 0.2083 | Yes |
| 18 | PLA2G1B | na | 1671 | 0.371 | 0.2170 | Yes |
| 19 | CACNA1E | na | 1688 | 0.370 | 0.2267 | Yes |
| 20 | MAPT | na | 1744 | 0.365 | 0.2352 | Yes |
| 21 | PRKACG | na | 1784 | 0.361 | 0.2441 | Yes |
| 22 | PLA2G2C | na | 2003 | 0.344 | 0.2472 | Yes |
| 23 | PRKCB | na | 2281 | 0.324 | 0.2481 | Yes |
| 24 | CACNG3 | na | 2317 | 0.322 | 0.2560 | Yes |
| 25 | FGF4 | na | 2628 | 0.302 | 0.2553 | Yes |
| 26 | PLA2G2A | na | 2876 | 0.287 | 0.2561 | Yes |
| 27 | MAP3K12 | na | 2896 | 0.286 | 0.2634 | Yes |
| 28 | CACNG4 | na | 2923 | 0.284 | 0.2705 | Yes |
| 29 | TGFB1 | na | 3047 | 0.278 | 0.2746 | Yes |
| 30 | NFATC4 | na | 3293 | 0.266 | 0.2749 | Yes |
| 31 | MAP2K6 | na | 3338 | 0.263 | 0.2809 | Yes |
| 32 | CACNB4 | na | 3383 | 0.261 | 0.2868 | Yes |
| 33 | PDGFB | na | 3642 | 0.250 | 0.2862 | Yes |
| 34 | FLNA | na | 4103 | 0.232 | 0.2793 | Yes |
| 35 | MAP2K1 | na | 4240 | 0.228 | 0.2816 | Yes |
| 36 | FGF23 | na | 4389 | 0.222 | 0.2834 | Yes |
| 37 | PAK1 | na | 4910 | 0.204 | 0.2740 | Yes |
| 38 | DUSP4 | na | 4951 | 0.203 | 0.2784 | Yes |
| 39 | CACNG6 | na | 4974 | 0.202 | 0.2834 | Yes |
| 40 | MAP3K14 | na | 4980 | 0.202 | 0.2888 | Yes |
| 41 | PTPN7 | na | 5238 | 0.194 | 0.2867 | Yes |
| 42 | MAP4K1 | na | 5622 | 0.182 | 0.2806 | Yes |
| 43 | DUSP5 | na | 5691 | 0.180 | 0.2836 | Yes |
| 44 | CDC25B | na | 5700 | 0.179 | 0.2883 | Yes |
| 45 | RPS6KA1 | na | 5757 | 0.178 | 0.2916 | Yes |
| 46 | CRKL | na | 5867 | 0.175 | 0.2933 | Yes |
| 47 | CACNA1H | na | 5961 | 0.172 | 0.2953 | Yes |
| 48 | HSPA6 | na | 5975 | 0.172 | 0.2997 | Yes |
| 49 | CACNB3 | na | 6009 | 0.171 | 0.3034 | Yes |
| 50 | IL1B | na | 6104 | 0.168 | 0.3053 | Yes |
| 51 | RELA | na | 6248 | 0.164 | 0.3057 | Yes |
| 52 | DUSP7 | na | 6271 | 0.163 | 0.3096 | Yes |
| 53 | DUSP2 | na | 6328 | 0.161 | 0.3124 | Yes |
| 54 | SRF | na | 6509 | 0.157 | 0.3115 | Yes |
| 55 | NFKB2 | na | 6590 | 0.155 | 0.3134 | Yes |
| 56 | MAPK12 | na | 6613 | 0.154 | 0.3170 | Yes |
| 57 | FGF13 | na | 6783 | 0.149 | 0.3162 | Yes |
| 58 | FGF6 | na | 6809 | 0.148 | 0.3196 | Yes |
| 59 | RASGRP4 | na | 7082 | 0.141 | 0.3156 | Yes |
| 60 | NTF3 | na | 7205 | 0.138 | 0.3159 | Yes |
| 61 | PPP3CC | na | 7238 | 0.137 | 0.3187 | Yes |
| 62 | FAS | na | 7283 | 0.136 | 0.3212 | Yes |
| 63 | NTF4 | na | 7333 | 0.135 | 0.3235 | Yes |
| 64 | MAP3K5 | na | 7445 | 0.132 | 0.3239 | Yes |
| 65 | STMN1 | na | 7518 | 0.130 | 0.3254 | Yes |
| 66 | DUSP8 | na | 7608 | 0.128 | 0.3264 | Yes |
| 67 | NGF | na | 7651 | 0.127 | 0.3287 | Yes |
| 68 | NRAS | na | 7693 | 0.126 | 0.3309 | Yes |
| 69 | ATF2 | na | 7864 | 0.121 | 0.3294 | Yes |
| 70 | PRKCA | na | 7938 | 0.120 | 0.3305 | Yes |
| 71 | MAP4K3 | na | 8096 | 0.117 | 0.3292 | Yes |
| 72 | GADD45A | na | 8193 | 0.114 | 0.3296 | Yes |
| 73 | GADD45B | na | 8406 | 0.110 | 0.3265 | Yes |
| 74 | RRAS | na | 8436 | 0.109 | 0.3287 | Yes |
| 75 | CACNA1A | na | 8517 | 0.108 | 0.3293 | Yes |
| 76 | TGFB3 | na | 8550 | 0.107 | 0.3313 | Yes |
| 77 | NFKB1 | na | 8680 | 0.105 | 0.3305 | Yes |
| 78 | CACNA1S | na | 8751 | 0.103 | 0.3313 | Yes |
| 79 | FGFR2 | na | 8886 | 0.101 | 0.3302 | Yes |
| 80 | MAPK8IP2 | na | 8887 | 0.101 | 0.3330 | Yes |
| 81 | CRK | na | 8997 | 0.099 | 0.3326 | Yes |
| 82 | MAP3K8 | na | 9005 | 0.099 | 0.3351 | Yes |
| 83 | GADD45G | na | 9055 | 0.098 | 0.3364 | Yes |
| 84 | PTPN5 | na | 9132 | 0.097 | 0.3369 | Yes |
| 85 | LAMTOR3 | na | 9277 | 0.094 | 0.3353 | Yes |
| 86 | MAP4K4 | na | 9378 | 0.092 | 0.3349 | Yes |
| 87 | PAK2 | na | 9466 | 0.091 | 0.3349 | Yes |
| 88 | MAP3K13 | na | 9481 | 0.091 | 0.3370 | Yes |
| 89 | FGF1 | na | 9677 | 0.087 | 0.3337 | No |
| 90 | RRAS2 | na | 9773 | 0.085 | 0.3333 | No |
| 91 | MAPKAPK2 | na | 9943 | 0.082 | 0.3307 | No |
| 92 | MAPK8IP1 | na | 10342 | 0.076 | 0.3212 | No |
| 93 | GRB2 | na | 10441 | 0.075 | 0.3205 | No |
| 94 | PTPRR | na | 10592 | 0.072 | 0.3181 | No |
| 95 | MAP4K2 | na | 10609 | 0.072 | 0.3196 | No |
| 96 | FLNC | na | 10616 | 0.072 | 0.3214 | No |
| 97 | RPS6KA6 | na | 10771 | 0.070 | 0.3189 | No |
| 98 | DUSP14 | na | 11137 | 0.064 | 0.3100 | No |
| 99 | DUSP16 | na | 11241 | 0.063 | 0.3088 | No |
| 100 | RASA2 | na | 11447 | 0.060 | 0.3045 | No |
| 101 | PPM1A | na | 11502 | 0.059 | 0.3046 | No |
| 102 | PPP5C | na | 11579 | 0.058 | 0.3039 | No |
| 103 | PPP3CA | na | 11626 | 0.057 | 0.3042 | No |
| 104 | TGFBR1 | na | 11693 | 0.057 | 0.3038 | No |
| 105 | TNFRSF1A | na | 11768 | 0.055 | 0.3032 | No |
| 106 | PPP3CB | na | 11848 | 0.055 | 0.3024 | No |
| 107 | ECSIT | na | 11939 | 0.053 | 0.3013 | No |
| 108 | FGF21 | na | 12140 | 0.051 | 0.2969 | No |
| 109 | MAP3K7 | na | 12201 | 0.050 | 0.2965 | No |
| 110 | PRKACA | na | 12347 | 0.048 | 0.2936 | No |
| 111 | EGFR | na | 12485 | 0.046 | 0.2910 | No |
| 112 | BDNF | na | 12595 | 0.045 | 0.2890 | No |
| 113 | PRKX | na | 12697 | 0.044 | 0.2873 | No |
| 114 | GNG12 | na | 12795 | 0.043 | 0.2857 | No |
| 115 | MAP3K2 | na | 12808 | 0.042 | 0.2865 | No |
| 116 | FGF14 | na | 12830 | 0.042 | 0.2870 | No |
| 117 | CACNB2 | na | 12833 | 0.042 | 0.2881 | No |
| 118 | ARRB2 | na | 12937 | 0.041 | 0.2863 | No |
| 119 | GNA12 | na | 13021 | 0.040 | 0.2850 | No |
| 120 | IL1A | na | 13065 | 0.040 | 0.2848 | No |
| 121 | FGF2 | na | 13152 | 0.039 | 0.2834 | No |
| 122 | DUSP6 | na | 13326 | 0.037 | 0.2794 | No |
| 123 | MOS | na | 13525 | 0.035 | 0.2746 | No |
| 124 | MAPKAPK5 | na | 13583 | 0.035 | 0.2739 | No |
| 125 | TRAF6 | na | 13633 | 0.034 | 0.2735 | No |
| 126 | EGF | na | 13899 | 0.031 | 0.2666 | No |
| 127 | RAC2 | na | 13916 | 0.031 | 0.2670 | No |
| 128 | HSPA1L | na | 14016 | 0.030 | 0.2650 | No |
| 129 | CD14 | na | 14336 | 0.027 | 0.2565 | No |
| 130 | TNF | na | 14399 | 0.027 | 0.2554 | No |
| 131 | MAPK1 | na | 14536 | 0.025 | 0.2522 | No |
| 132 | HRAS | na | 14559 | 0.025 | 0.2523 | No |
| 133 | CHP1 | na | 14674 | 0.024 | 0.2496 | No |
| 134 | TP53 | na | 14698 | 0.024 | 0.2496 | No |
| 135 | FGF12 | na | 14791 | 0.023 | 0.2476 | No |
| 136 | RELB | na | 15014 | 0.021 | 0.2417 | No |
| 137 | RAF1 | na | 15219 | 0.019 | 0.2363 | No |
| 138 | PLA2G4A | na | 15227 | 0.019 | 0.2366 | No |
| 139 | STK3 | na | 15282 | 0.019 | 0.2356 | No |
| 140 | MAP2K5 | na | 15356 | 0.018 | 0.2339 | No |
| 141 | PPP3R1 | na | 15411 | 0.018 | 0.2329 | No |
| 142 | MAP2K3 | na | 15568 | 0.016 | 0.2288 | No |
| 143 | IKBKG | na | 15619 | 0.016 | 0.2278 | No |
| 144 | AKT1 | na | 15787 | 0.014 | 0.2233 | No |
| 145 | PLA2G6 | na | 15813 | 0.014 | 0.2230 | No |
| 146 | FGF10 | na | 16172 | 0.011 | 0.2129 | No |
| 147 | RAC1 | na | 16331 | 0.010 | 0.2086 | No |
| 148 | TAB2 | na | 16358 | 0.010 | 0.2081 | No |
| 149 | FGF19 | na | 16438 | 0.009 | 0.2061 | No |
| 150 | DUSP1 | na | 16530 | 0.008 | 0.2037 | No |
| 151 | STK4 | na | 16565 | 0.008 | 0.2029 | No |
| 152 | JUN | na | 16587 | 0.008 | 0.2026 | No |
| 153 | MAPK3 | na | 16821 | 0.006 | 0.1960 | No |
| 154 | HSPB1 | na | 16884 | 0.006 | 0.1943 | No |
| 155 | RAP1A | na | 16926 | 0.005 | 0.1933 | No |
| 156 | FGF11 | na | 16966 | 0.005 | 0.1923 | No |
| 157 | MAPK8 | na | 17140 | 0.003 | 0.1873 | No |
| 158 | ATF4 | na | 17294 | 0.002 | 0.1829 | No |
| 159 | JUND | na | 17322 | 0.002 | 0.1822 | No |
| 160 | FGF16 | na | 17343 | 0.001 | 0.1817 | No |
| 161 | DAXX | na | 17402 | 0.001 | 0.1800 | No |
| 162 | FGF22 | na | 17511 | 0.000 | 0.1769 | No |
| 163 | MAPK7 | na | 17787 | -0.002 | 0.1690 | No |
| 164 | CDC42 | na | 17874 | -0.003 | 0.1665 | No |
| 165 | FGF20 | na | 17878 | -0.003 | 0.1665 | No |
| 166 | RAP1B | na | 18052 | -0.005 | 0.1616 | No |
| 167 | RAPGEF2 | na | 18152 | -0.005 | 0.1589 | No |
| 168 | PLA2G12A | na | 18221 | -0.006 | 0.1571 | No |
| 169 | PLA2G2E | na | 18403 | -0.008 | 0.1521 | No |
| 170 | MAP2K7 | na | 18710 | -0.010 | 0.1435 | No |
| 171 | TAB1 | na | 18745 | -0.011 | 0.1428 | No |
| 172 | MAP3K1 | na | 18900 | -0.012 | 0.1386 | No |
| 173 | MAP3K11 | na | 19010 | -0.013 | 0.1358 | No |
| 174 | FGF7 | na | 19070 | -0.014 | 0.1345 | No |
| 175 | NLK | na | 19287 | -0.015 | 0.1286 | No |
| 176 | MAX | na | 19465 | -0.017 | 0.1240 | No |
| 177 | MAPKAPK3 | na | 19593 | -0.018 | 0.1208 | No |
| 178 | TRAF2 | na | 19649 | -0.018 | 0.1197 | No |
| 179 | MYC | na | 19651 | -0.019 | 0.1202 | No |
| 180 | TGFBR2 | na | 20164 | -0.023 | 0.1060 | No |
| 181 | CHUK | na | 20245 | -0.024 | 0.1043 | No |
| 182 | MAP2K2 | na | 20653 | -0.028 | 0.0933 | No |
| 183 | RPS6KA3 | na | 20863 | -0.030 | 0.0881 | No |
| 184 | TAOK3 | na | 20971 | -0.031 | 0.0858 | No |
| 185 | MAPK10 | na | 20989 | -0.032 | 0.0862 | No |
| 186 | MAP3K3 | na | 21084 | -0.032 | 0.0844 | No |
| 187 | CACNG1 | na | 21206 | -0.034 | 0.0818 | No |
| 188 | MAP2K4 | na | 21265 | -0.035 | 0.0811 | No |
| 189 | TAOK1 | na | 21270 | -0.035 | 0.0819 | No |
| 190 | HSPA1A | na | 21593 | -0.038 | 0.0736 | No |
| 191 | MKNK2 | na | 21890 | -0.042 | 0.0662 | No |
| 192 | DUSP3 | na | 21902 | -0.042 | 0.0670 | No |
| 193 | CACNA1C | na | 22079 | -0.044 | 0.0631 | No |
| 194 | PDGFA | na | 22257 | -0.046 | 0.0592 | No |
| 195 | HSPA1B | na | 22392 | -0.047 | 0.0566 | No |
| 196 | CACNG5 | na | 22404 | -0.047 | 0.0576 | No |
| 197 | SOS2 | na | 22550 | -0.049 | 0.0548 | No |
| 198 | RPS6KA4 | na | 22603 | -0.050 | 0.0546 | No |
| 199 | ELK4 | na | 22798 | -0.052 | 0.0504 | No |
| 200 | NF1 | na | 22940 | -0.054 | 0.0478 | No |
| 201 | PPM1B | na | 23099 | -0.056 | 0.0448 | No |
| 202 | MAPK14 | na | 23324 | -0.059 | 0.0399 | No |
| 203 | IKBKB | na | 23356 | -0.060 | 0.0407 | No |
| 204 | JMJD7-PLA2G4B | na | 23573 | -0.063 | 0.0362 | No |
| 205 | FGFR4 | na | 23580 | -0.063 | 0.0378 | No |
| 206 | MAPK8IP3 | na | 23928 | -0.069 | 0.0296 | No |
| 207 | MAPK13 | na | 23983 | -0.069 | 0.0299 | No |
| 208 | PLA2G3 | na | 24134 | -0.071 | 0.0275 | No |
| 209 | MAP3K6 | na | 24443 | -0.076 | 0.0207 | No |
| 210 | HSPA2 | na | 24524 | -0.077 | 0.0205 | No |
| 211 | MKNK1 | na | 24663 | -0.080 | 0.0187 | No |
| 212 | FGFR3 | na | 24695 | -0.080 | 0.0200 | No |
| 213 | MAPK9 | na | 24826 | -0.082 | 0.0186 | No |
| 214 | CASP3 | na | 24828 | -0.083 | 0.0208 | No |
| 215 | TGFB2 | na | 24944 | -0.084 | 0.0198 | No |
| 216 | BRAF | na | 25048 | -0.086 | 0.0192 | No |
| 217 | KRAS | na | 25083 | -0.087 | 0.0206 | No |
| 218 | FGF8 | na | 25299 | -0.091 | 0.0168 | No |
| 219 | MAP3K4 | na | 25312 | -0.091 | 0.0190 | No |
| 220 | NTRK1 | na | 25343 | -0.091 | 0.0206 | No |
| 221 | MAPK11 | na | 25479 | -0.094 | 0.0193 | No |
| 222 | RASA1 | na | 25797 | -0.100 | 0.0129 | No |
| 223 | HSPA8 | na | 25938 | -0.103 | 0.0117 | No |
| 224 | PLA2G5 | na | 25954 | -0.103 | 0.0141 | No |
| 225 | RPS6KA5 | na | 26273 | -0.110 | 0.0079 | No |
| 226 | CACNA2D3 | na | 26367 | -0.112 | 0.0083 | No |
| 227 | CACNA1D | na | 26374 | -0.112 | 0.0112 | No |
| 228 | RASGRP2 | na | 26393 | -0.113 | 0.0138 | No |
| 229 | TAOK2 | na | 26642 | -0.118 | 0.0098 | No |
| 230 | SOS1 | na | 26852 | -0.122 | 0.0071 | No |
| 231 | FLNB | na | 26864 | -0.122 | 0.0102 | No |
| 232 | CACNG2 | na | 26917 | -0.123 | 0.0121 | No |
| 233 | PLA2G10 | na | 27060 | -0.127 | 0.0115 | No |
| 234 | CACNA1F | na | 27070 | -0.127 | 0.0147 | No |
| 235 | AKT2 | na | 27111 | -0.128 | 0.0171 | No |
| 236 | NR4A1 | na | 27229 | -0.131 | 0.0173 | No |
| 237 | RPS6KA2 | na | 27234 | -0.132 | 0.0208 | No |
| 238 | PLA2G4E | na | 27235 | -0.132 | 0.0245 | No |
| 239 | PLA2G2F | na | 27530 | -0.139 | 0.0198 | No |
| 240 | FGF3 | na | 27771 | -0.145 | 0.0168 | No |
| 241 | FOS | na | 27908 | -0.149 | 0.0170 | No |
| 242 | CACNG7 | na | 28246 | -0.158 | 0.0115 | No |
| 243 | RASGRF1 | na | 28263 | -0.158 | 0.0154 | No |
| 244 | DUSP9 | na | 28445 | -0.163 | 0.0147 | No |
| 245 | PLA2G2D | na | 28487 | -0.164 | 0.0180 | No |
| 246 | CHP2 | na | 28497 | -0.164 | 0.0223 | No |
| 247 | MECOM | na | 28559 | -0.166 | 0.0251 | No |
| 248 | PRKCG | na | 28978 | -0.178 | 0.0179 | No |
| 249 | FASLG | na | 29443 | -0.193 | 0.0097 | No |
| 250 | ARRB1 | na | 29554 | -0.196 | 0.0120 | No |
| 251 | NFATC2 | na | 29713 | -0.201 | 0.0129 | No |
| 252 | FGF9 | na | 30736 | -0.238 | -0.0102 | No |
| 253 | ELK1 | na | 30931 | -0.244 | -0.0091 | No |
| 254 | CACNB1 | na | 31307 | -0.259 | -0.0128 | No |
| 255 | CACNG8 | na | 31602 | -0.274 | -0.0138 | No |
| 256 | FGF18 | na | 32061 | -0.297 | -0.0189 | No |
| 257 | CACNA1I | na | 32230 | -0.308 | -0.0152 | No |
| 258 | RAC3 | na | 32406 | -0.320 | -0.0115 | No |
| 259 | FGF17 | na | 32598 | -0.334 | -0.0078 | No |
| 260 | PPP3R2 | na | 32636 | -0.337 | 0.0004 | No |
| 261 | FGF5 | na | 32744 | -0.346 | 0.0069 | No |
| 262 | PDGFRB | na | 32781 | -0.349 | 0.0155 | No |
| 263 | PLA2G12B | na | 33562 | -0.422 | 0.0045 | No |
| 264 | PDGFRA | na | 33905 | -0.477 | 0.0077 | No |
| 265 | RASGRF2 | na | 34331 | -0.584 | 0.0115 | No |
Table: GSEA details [plain text format]

  

Fig 2: KEGG\_MAPK\_SIGNALING\_PATHWAY      
 Blue-Pink O' Gram in the Space of the Analyzed GeneSet

  

Fig 3: KEGG\_MAPK\_SIGNALING\_PATHWAY: Random ES distribution      
 Gene set null distribution of ES for **KEGG\_MAPK\_SIGNALING\_PATHWAY**
